# Saliva liquid biopsy: MMP2, MMP9, and TIMP2 as potential diagnostic biomarkers in oral squamous cell carcinoma. An observational case-control study

**DOI:** 10.1590/1678-7765-2025-0555

**Published:** 2026-03-02

**Authors:** Sergio D. Cruz-Romero, Ana Margarita Baldión, José Antonio Hakim Tawil, Alberto Escallon, Paula A. Rodriguez-Urrego, Paula Alejandra Baldión

**Affiliations:** 1 Universidad Nacional de Colombia Facultad de Odontologia Departamento de Salud Oral Bogotá Colombia Universidad Nacional de Colombia, Facultad de Odontologia, Departamento de Salud Oral, Bogotá, Colombia; 2 Hospital Universitario Fundación Santa Fe de Bogotá Departamento de Patologia y Laboratorios Bogotá Colombia Hospital Universitario Fundación Santa Fe de Bogotá, Departamento de Patologia y Laboratorios, Bogotá, Colombia; 3 Hospital Universitario Fundación Santa Fe de Bogotá Cirugia de Cabeza y Cuello Bogotá Colombia Hospital Universitario Fundación Santa Fe de Bogotá, Cirugia de Cabeza y Cuello, Bogotá, Colombia

**Keywords:** Squamous cell carcinoma of head and neck, Matrix metalloproteinases, Tissue inhibitor of metalloproteinase-2, Saliva

## Abstract

Oral squamous cell carcinoma (OSCC) is the most common malignant neoplasm in the oral cavity, characterized by high morbidity and mortality. Various proteins, including matrix metalloproteinases (MMPs), have been investigated as potential biomarkers for diagnosing and prognosing this disease. Objective: To evaluate the utility of MMP-2, MMP-9, and the tissue inhibitor of metalloproteinase-2 (TIMP-2) as biomarkers in diagnosing and prognosing OSCC. Methodology: This retrospective case-control study involved 60 cases and 30 controls from the University Hospital Fundación Santa Fe de Bogotá, part of the Colombian cohort of the InterChange/Headspace study. Saliva samples were collected using a 60-second mouth rinse with 10 mL of sterile saline solution, along with serum and oral tissue samples and clinical and sociodemographic data. MMP-9, MMP-2, and TIMP-2 concentrations in saliva were determined using ELISA, whereas tissue samples were analyzed by immunohistochemistry. MMP activity in saliva was quantified using a generic activity assay. Results: Salivary MMP-9 and MMP-2 concentrations were significantly higher in cases than in controls, whereas no significant differences were observed for TIMP-2. ROC curve analysis showed excellent discriminatory capacity for salivary MMP-9 (AUC=0.946; sensitivity=0.817; specificity=0.867), good performance for MMP-2 (AUC=0.708; sensitivity=0.717; specificity=0.633), and lower discriminatory ability for TIMP-2 (AUC=0.630; sensitivity = 0.617; specificity=0.500). Elevated MMP-9 concentrations were observed in 33% of the serological samples. The immunohistochemistry of MMP-2, MMP-9, and TIMP-2 showed differences between cases and controls. Conclusions: This study highlights salivary MMP-9 and MMP-2, together with MMP activity, as biomarkers of interest for OSCC that may contribute to the understanding of disease-related molecular changes detectable by liquid biopsy. While elevated salivary levels were observed in OSCC cases when compared to controls, these findings should be interpreted as exploratory. Elevated serological MMP-9 levels were observed in a small subset of cases. However, the limited sample size and the absence of a control group in this study preclude diagnostic interpretation. Larger, well-powered, and externally validated studies are required to determine the potential clinical utility of these biomarkers.

## Graphical abstract

**Figure:**
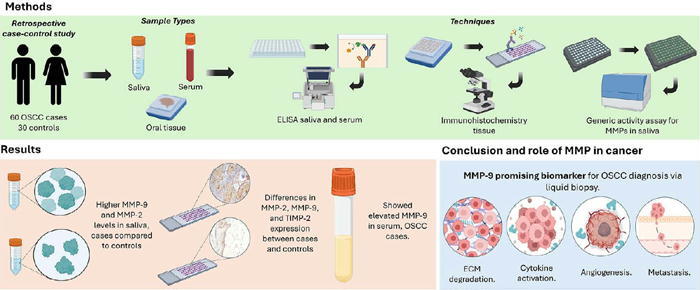

This manuscript is derived from the thesis Salivary levels of MMP2, MMP9, and TIMP2 as potential diagnostic biomarkers in oral squamous cell carcinoma, available at: https://repositorio.unal.edu.co/items/9e7505ba-4b0c-4485-bcf5-abd55290ab6f.

## Introduction

Oral squamous cell carcinoma (OSCC) is the most prevalent malignant neoplasm of the oral cavity, with a global incidence of 377,713 cases and a mortality rate of 177,757 people in 2020.^1^ The disease predominantly affects men aged over 40 years, particularly those with a history of smoking for more than 10 years, alcohol consumption, and exposure to other associated risk factors.^2^, ^3^, ^4^

Histologically, OSCC arises from the squamous epithelial cells lining the oral cavity. It can develop in various subsites, including the lips, buccal mucosa, hard palate, anterior tongue, floor of the mouth, and retromolar trigone.^2^ The morbidity associated with OSCC is significant as the disease frequently involves the orofacial region and cervical lymph node dissemination as well as lymphatic involvement in advanced stages.^2^, ^3^

Numerous biomarkers have been investigated for their prognostic value in OSCC, including nucleic acids, peptides, proteins, and metabolic intermediates.^5^ Among these, matrix metalloproteinases (MMPs) have gained attention due to their critical role in tumor progression.^6^, ^7^ MMPs are zinc-dependent endopeptidases that contribute to the remodeling of the extracellular matrix (ECM) under physiological conditions and regulate various cellular interactions in response to environmental cues.^8^, ^9^ In pathological conditions, particularly in solid organ carcinomas, MMPs alter the tumor microenvironment, promoting tumor growth and metastasis.^10^

MMP-9 and MMP-2 have been implicated in the pathogenesis of OSCC due to their ability to degrade key components of the ECM (such as collagen, elastin, and fibronectin) and their role in angiogenesis regulation.^11^ These proteins have been studied as prognostic markers in OSCC, with immunohistochemical analyses showing higher expression of MMP-9 and MMP-2 in tumor tissues when compared to normal tissues. This overexpression correlates with adverse prognostic factors, including advanced clinical stages, high histological grades, lymph node metastasis, and tumor recurrence.^12^, ^13^

In addition to tissue-based studies, molecular markers such as MMPs have also been explored in human fluids, including serum, saliva, plasma, and urine—a technique known as liquid biopsy.^14^ Saliva has shown promise as a non-invasive sample for detecting diagnostic, prognostic, and monitoring biomarkers in OSCC due to its direct contact with the oral environment and the metabolic activity of tumor lesions.^15^ Serum, among other fluids, has also been studied for its clinical relevance in OSCC.^16^

Research has found higher concentrations of MMP-9 in the saliva of OSCC patients than in healthy controls, underscoring the potential of salivary biomarkers in diagnosing and prognosing this disease.^17^ However, our review found a lack of experimental studies focusing on MMP-2, MMP-9, and tissue inhibitor of metalloproteinase-2 (TIMP-2) as salivary biomarkers in a Colombian cohort, highlighting a critical gap in the literature.

Given the potential of MMPs as prognostic biomarkers, this study aims to assess the utility of salivary liquid biopsy to detect MMP-2, MMP-9, and TIMP-2 in the diagnosis of OSCC in a Colombian cohort. Additionally, it will compare these salivary biomarkers with tissue expression by immunohistochemistry and their concentration in blood by ELISA.

## Methodology

### Study design

An analytical observational retrospective case-control study was conducted with patients from the Colombian cohort of the InterChange/HeadSpace study at Fundación Santa Fe de Bogotá from 2015 to 2022.

### Cases and controls

The case group consisted of patients with histopathologically confirmed OSCC diagnosed in their buccal mucosa, palate, tongue, or cheeks at Fundación Santa Fe de Bogotá. All cases were newly diagnosed, with a maximum interval of three months between diagnosis and enrollment.

The control group was selected according to the criteria established by the InterChange/HeadSpace study, a protocol designed to create a biorepository of biological samples for multiple research purposes (rather than specifically for the present analysis). Controls were matched to cases by sex and age (± 5 years), with a ratio of 1:2 for saliva samples. A convenience sampling strategy was used.

Controls were recruited from hospitalized patients with recently diagnosed non-chronic conditions with no association with alcohol or tobacco use, verified by clinical records. Controls were excluded—rather than based on alcohol or tobacco consumption itself—by the presence of chronic diseases associated with these habits to minimize confounding related to systemic inflammatory or metabolic conditions. Exclusion criteria for controls included a history of head and neck cancer, severe psychiatric disorders, cognitive impairment, neurodegenerative diseases, acute cerebrovascular events, advanced tumors, brain metastases, or clinical conditions that could compromise data quality or questionnaire reliability.

All participants provided written informed consent. Cases consented to the collection of saliva, blood, and tumor tissue samples, whereas controls consented only to saliva and blood collection (as no tissue samples were obtained from controls). This study complied with the Declaration of Helsinki and received ethical approval from Fundación Santa Fe de Bogotá (CCEI-2424-2014) and the Faculty of Dentistry at Universidad Nacional de Colombia (B.CIEFO-112-2023).

### Sample collection and data acquisition

For the case group, tissue samples were collected at the time of diagnosis before any therapeutic intervention. Saliva samples were obtained using a saline mouthwash method, ensuring the collection of cells and proteins from the oral cavity. Moreover, clinical data (including medical history and lifestyle factors) were obtained by patient records and structured surveys. Follow-up data were gathered to assess the outcomes and progression of the disease.

For the control group, saliva samples and lifestyle data were collected similarly, following the same protocol used for the cases. Controls were followed up to confirm the absence of OSCC or other significant oral pathology.

### Biomarker detection by enzyme-linked immunosorbent assay (ELISA)

Saliva samples were collected using a 60-second mouth rinse with 10 mL of sterile 0.9% saline solution, which was then collected in 50 mL tubes. These samples were stored and transported at 4 °C for no longer than 3 hours before being centrifuged at 2500 rpm for 10 minutes at 4 °C. Following centrifugation, 4 mL of the supernatant was carefully transferred into the 1.8 mL cryotubes and stored at -80 °C until analysis. This protocol was consistently applied to samples from patients and healthy controls.

Blood samples were drawn into 5-mL SST tubes and stored at 4 °C for no more than 2 hours before processing. The blood was then centrifuged at 3500 rpm for 10 minutes at 4 °C. Then, two serum aliquots were transferred into cryotubes and stored at −80 °C until biomarker detection. The samples were stored for an average of three years.

Salivary concentrations of MMP-2, MMP-9, and TIMP-2 were quantified using specific ELISA kits for each analyte (Merck KGaA, Darmstadt, Germany), following the manufacturer’s protocols. Standard curves were established by serial dilutions of each protein concentrate. All assays were run in duplicates. For each sample, 100 μL were added to a 96-well plate and incubated at room temperature for 2.5 hours. After incubation, the excess solution was removed and the wells were washed with 300 μL of washing buffer, repeating the wash steps as required. Detection antibodies (100 μL) were added to each well, followed by a one-hour incubation at room temperature with gentle shaking. Streptavidin conjugated with horseradish peroxidase was then added and incubated for 45 minutes. Following this, 100 μL of a 3,3’,5,5’-tetramethylbenzidine substrate solution was applied, and the reaction was developed in the dark at room temperature for 30 minutes.

**Figure 1:**
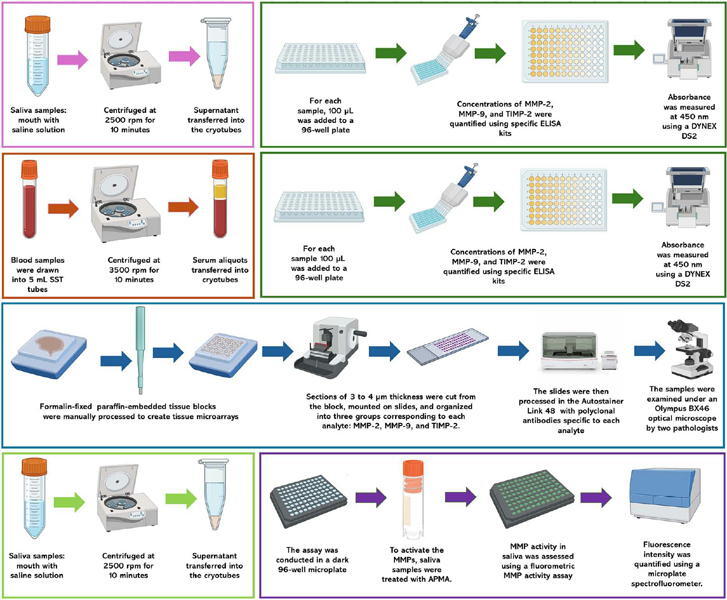
Schematic representation of the methodological workflow.

Finally, to stop the reaction, 100 μL of a stop solution was added to each well, and absorbance was measured at 450 nm using a DYNEX DS2 ELISA plate reader (Dynex, VA, USA). Concentrations were determined based on absorbance values using the Dynex DS2 2-Plate ELISA Processing System software (Dynex). Results for MMP-9 and TIMP-2 were first obtained in pg/mL (as indicated by the ELISA plate format) and then converted to ng/mL to enable comparisons with MMP-2 and the values in the literature. MMP-2 concentrations were measured directly in ng/mL. This conversion was applied uniformly across all samples to ensure consistency in data description (Figure 1).

### Histopathological analysis

Tissue samples were obtained either by biopsy or surgical resection, depending on availability. The samples were fixed in 10% paraffin-embedded formaldehyde and cut at 4-μm slices using an HM355 microtome (Thermo Fisher Scientific, MA, USA). The sections were mounted on slides and stained with hematoxylin-eosin.

A specialized head and neck pathologist conducted the histopathological examination, confirming the OSCC diagnosis and assessing key histopathological parameters. These parameters included tumor histological classification, size, histological grade, depth of infiltration, lymphovascular invasion, perineural invasion, margins, bone involvement, lymph node metastasis, and TNM classification following the guidelines of the American Joint Committee on Cancer 8th edition.^18^

### Clinical and sociodemographic factors

Clinical and follow-up data were extracted from patients’ medical records, including treatment modalities (surgical, chemotherapy, radiotherapy, immunotherapy), treatment response, recurrence, metastasis, and cancer-associated mortality.

Sociodemographic information was obtained from the lifestyle questionnaire in the Interchange/Headspace study. These data, collected by identical interviews with all cases and controls, included demographic details (age, sex, ethnicity, residence, education), smoking history, alcohol consumption, and oral health status.

### Detection of biomarkers in oral tissue

A total of 46 paraffin-embedded formaldehyde tissue blocks were manually processed to create tissue microarrays. A pathologist identified and marked representative tumor areas on hematoxylineosin slides. From these marked regions, 4-mm diameter tissue cores were extracted using a cylindrical punch and embedded into a recipient block containing cores from various cases. Moreover, normal adjacent tissue samples were obtained from the same biopsies or surgical resections of patients with OSCC. These specimens corresponded to histologically normal mucosa located at least 1 cm from the tumor margins and confirmed as free of neoplastic infiltration by a head and neck pathologist. This normal adjacent tissue was used as the control group for immunohistochemical comparisons. Sections of 3 to 4 μm thickness were cut from each block, mounted on slides, and organized into three groups corresponding to each analyte: MMP-2, MMP-9, and TIMP-2.

The tissue microarray slides were deparaffinized under dry heat at 62 °C for 1 hour, followed by heat-induced antigen retrieval at 92 °C with DakoTarget Retrieval Solution (pH 9, 10X) (Agilent Technologies, CA, USA) for 40 mn using the Dako PTLink system (Agilent Technologies). The slides were then processed in the Autostainer Link 48 (Agilent Technologies) for immunostaining. Endogenous peroxidase activity was blocked with EnVision FLEX Peroxidase-Blocking Reagent (SM801, Agilent Technologies) for 7 mn. Nonspecific binding sites were blocked by incubation in phosphate-buffered saline with 1% casein (C5890, Merck KGaA) for 10 mn. The slides were then incubated with rabbit polyclonal primary antibodies specific to each analyte: anti-MMP-9 (ab38898, Abcam, MA, USA) for 20 mn, anti-MMP-2 (ab97779, Abcam) for 25 mn, and anti-TIMP-2 (ab180630, Abcam) for 30 mn. The Envision FLEX horseradish peroxidase detection system (Agilent Technologies) was used for primary antibody detection, followed by immunostaining with the EnVision FLEX DAB+ Substrate Chromogen System (Agilent Technologies). Mayer’s hematoxylin was applied for counterstaining.

Positive controls included endometrial carcinoma tissue for MMP-2 and colon carcinoma tissue for TIMP-2 and MMP-9. Negative controls were performed by omitting the primary antibody. After immunostaining, the samples were examined under an Olympus BX46 optical microscope (Olympus Corporation, Tokyo, Japan) by a blinded pathologist who was unaware of the clinical details. Intensity 2+, 3+) and percentage positivity (0% to 100%) were evaluated, with final scores calculated by multiplying intensity and percentage values, resulting in a final score ranging from 0 to 12^19^ (Figure 1).

### Matrix metalloproteinase activity assay in saliva

MMP activity in saliva liquid biopsies was assessed using a fluorometric MMP activity assay (Fluorometric: Green; Abcam). To activate the MMPs, saliva samples were treated with 4-aminophenylmercuric acetate (APMA, Abcam) for two hours prior to the assay. The assay was conducted in a dark 96-well microplate (Corning, NY, USA). Each well was incubated with a fluorogenic substrate—fluorescein isothiocyanate (Abcam)—for 30 minutes. This substrate uses fluorescence resonance energy transfer to indicate MMP activity. In the absence of protease activity, the fluorescence signals remain low due to quenching by the proximal fluorochrome. Conversely, when protease activity is present, the peptide spacer is cleaved, releasing the fluorochrome and producing a measurable fluorescent signal.

Fluorescence intensity was quantified using a microplate spectrofluorometer (Fluoroskan, Thermo Fisher Scientific) with excitation and emission filters set to 485/538 nm. Relative fluorescence units (RFU) were compared to a positive control, which consisted of an MMP standard at 50 ng/mL (Merck KGaA). Ethylenediaminetetraacetic acid at 1 mM was used as an inhibition control, being added 15 minutes before substrate addition to assess the specificity of MMP activity (Figure 1).

### Statistical analysis

Data were organized in an Excel spreadsheet (Microsoft Office 2010) with the appropriate variable coding. Statistical analyses were performed on Past 4.16 (University of Oslo, Norway). Descriptive statistics included absolute and relative frequencies for categorical variables and means ± standard deviation for continuous variables.

The Shapiro-Wilk test was used to assess data distribution normality. To compare protein concentrations in saliva between OSCC patients and healthy controls and the mean expression of markers by immunohistochemistry between cases and controls, the Mann-Whitney U test was employed. Serum biomarker concentrations were analyzed using the Kruskal-Wallis H test, followed by post hoc analysis with the Tukey’s test.

To explore associations between clinical and demographic characteristics and biomarkers, non-parametric tests (Kruskal-Wallis H, Mann-Whitney U, and Spearman’s Rho), were utilized due to the non-normal distribution of data.

Receiver operating characteristic (ROC) curves were generated to estimate the diagnostic value of each protein based on immunohistochemistry and to determine optimal cut-off points. Additional ROC curves were developed to evaluate the prognostic value of salivary biomarker concentrations and tissue immunohistochemical staining according to their sensitivity and specificity.

## Results

### Sociodemographic and clinical information

This study included 60 OSCC cases and 30 healthy controls. In the OSCC group, 58.33% (n = 35) were women and 41.67% (n = 25) were men, with a mean age of 66 years (±14). In the control group, the gender distribution was equal, with 50.00% (n=15) women and 50.00% (n=15) men. Their mean age was 61 years (±11) (Table 1).

**Table 1:** Sociodemographic, clinical, and histopathological characteristics of patients with oral squamous cell carcinoma (OSCC).

**Sociodemographic characteristics of cases**		
	**N**	**%**
**Gender**		
Female	35	58.33%
Male	25	41.67%
**Age**		
Mean age	66 (±14)	
**Smoker**		
No	35	58.33%
Yes	25	41.67%
**Smoking status**		
Never smoked	35	58.33%
Smoked in the past	25	41.67%
Currently smokes	0	0.00%
**Cigarettes a day**		
0 cigarettes a day	35	58.33%
1 to 4 cigarettes a day	10	16.67%
5 to 9 cigarettes a day	8	13.33%
10 or more cigarettes a day	7	11.67%
**Years of smoking**		
0 years smoking	35	58.33%
1 to 9 years smoking	5	8.33%
10 to 29 years smoking	14	23.33%
30 or more years of smoking	6	10.00%
**Alcohol consumption**		
No	30	50.00%
Yes	30	50.00%
Alcohol consumption status		
Currently consuming alcohol	18	30.00%
Consumed alcohol in the past	11	18.33%
Never consumed alcohol	31	51.67%
**Number of days a week consumes alcohol**		
0 days of the week	31	51.67%
1 to 2 days a week	19	40.00%
3 or more days a week	5	8.33%
**Drinks a day**		
0 drinks a day	31	51.67%
1 to 4 drinks a day	14	23.33%
5 to 9 drinks a day	8	13.33%
10 or more drinks a day	7	11.67%
**Clinical features of cases**		
	**N**	**%**
**Comorbidities**		
Without comorbidities	32	53.33%
With comorbidities	28	46.67%
**Recurrent cases**		
No recurrence	39	65.00%
Local recurrence	21	35.00%
**Death associated with disease**		
5-year survival	47	78.33%
5-year mortality	13	21.67%
**Surgical resection of the initial primary**		
No	3	5.00%
Yes	57	95.00%
**Surgical technique**		
Hemiglossectomy	12	20.00%
Hemiglossectomy plus neck dissection	36	60.00%
Mandibulectomy plus radical neck dissection	5	8.33%
Maxillofacial Surgery	1	1.67%
Maxillofacial surgery plus neck dissection	3	5.00%
**Radiotherapy**		
No	28	46.67%
Yes	32	53.33%
**Chemotherapy**		
No	43	71.67%
Yes	17	28.33%
**Chemotherapy regimen**		
Cisplatin	13	13
Carboplatin, cetuximab, docetaxel	1	1
Carboplatin, paclitaxel	1	1
Cetuximab	1	1
Pembrolizumab	1	1
n/a	43	43
**Histopathological characteristics of cases**		
	**N**	%
**Anatomical location cases**		
C02.9. Tongue	49	81.67%
C03.0 upper gingiva/ c03.1 lower gingiva	3	5.00%
C04.9 floor of the mouth	3	5.00%
C05.0 hard palate	3	5.00%
C06.0 cheek mucosa	2	3.33%
**Lymphovascular invasion cases**		
n/a	1	1.67%
No	52	86.67%
Yes	7	11.67%
**Perineural invasion cases**		
n/a	1	1.67%
No	28	46.67%
Yes	31	51.67%
**Histological grade**		
Well differentiated	29	48.33%
Moderately differentiated	21	35.00%
Poorly differentiated	10	16.67%
**Lymph node involvement**		
n/a	16	26.67%
Negative	22	36.67%
Positive	22	36.67%
**Clinical stage cases**		
I	20	33.33%
II	8	13.33%
III	9	15.00%
IVA	20	33.33%
IVB	2	3.33%
n/a	1	1.67%

Among the OSCC patients, 41.67% (n=25) were smokers. Of these, 25.00% (n=15) smoked more than five cigarettes per day, and 33.33% (n=18) had been smoking for over 10 years. Alcohol consumption was reported in 50.00% (n=30) of the OSCC group, with 30.00% (n=18) currently drinking and 18.33% (n=11) having consumed alcohol in the past. Additionally, 8.33% (n=5) drank alcohol more than three days a week, and 25.00% (n=15) consumed more than five drinks per day. Notably, 30.00% (n=18) of the OSCC patients were smokers and alcohol consumers (Table 1).

Treatment for OSCC included oncological resection in 95% of cases (n = 57), with 61.67% (n = 37) undergoing oncological resection and neck dissection. Intensity-modulated radiotherapy was administered to 53.33% (n=32) of the patients, and 28.33% (n=17) received chemotherapy, primarily platinum-based regimens (cisplatin): 21.67% (n=13) of cases.

All patients were followed up for five years. The five-year mortality rate totaled 16.67% and the recurrence rate, 31.67% (Table 1).

### Histological characteristics of the cases

**Figure 2:**
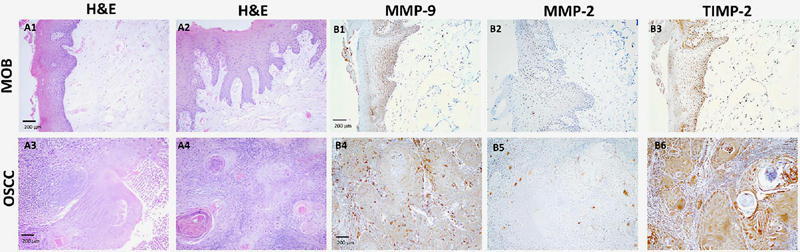
(A) Histopathological images stained with hematoxylin and eosin (H&E) and (B) Expression of MMP-9, MMP-2, and TIMP-2 by immunohistochemistry. Tissues were fixed on slides and stained with H&E. Expression was detected using primary antibodies specific to each analyte (Abcam). (A1-A2) Normal adjacent squamous mucosa used as control tissue, showing stratified squamous epithelium on the surface with underlying connective tissue from the posterior region of the oral tongue (100*). (A3-A4) Infiltrative squamous cell carcinoma, showing connective tissue invasion, inflammatory infiltrate, and keratin pearl formation (100*). (B1-B3) Immunohistochemical expression in normal adjacent mucosa (control): (B1) MMP-9, positive cytoplasmic staining in the basal epithelial layer (100*), (B2) MMP-2, weak (1+) cytoplasmic expression in the basal epithelial cells (100*), (B3) TIMP-2, positive expression in epithelial basal cells and connective tissue (100*). (B4-B6) Expression in tumor tissue: (B4) MMP-9, strong cytoplasmic expression in malignant epithelial cells (100*), (B5) MMP-2, weak cytoplasmic expression (1+) in tumor epithelial cells (40*), and (B6) TIMP-2, positive cytoplasmic staining in carcinoma cells and inflammatory infiltrate (100*). Scale bar: 200 μm.

Among the cases, 98.33% (n = 59) were classified as infiltrating keratinizing squamous cell carcinoma, whereas 1.67% (n=1) were diagnosed as verrucous carcinoma (Figure 2). According to the ICD-O3 coding, the tumors were primarily in the tongue (C02.9, 81.67%, n = 49), with other locations including the hard palate (C05.0, 5.00%, n=3), the floor of the mouth (C04.9, 5.00%, n = 3), the gums (C03.0-C03.1, 5.00%, n=3), and the cheek mucosa (C06.0, 3.33%, n = 2) (Table 1).

Lymph node involvement was noted in 36.67% (n = 22) of cases, with 16.67% (n=10) showing involvement of a single unilateral lymph node. Most carcinomas were classified as either stage I (33.33%) or IV (33.33%) (Table 1).

### Salivary concentrations of MMP-9, MMP-2, and TIMP-2 in cases and controls

**Figure 3:**
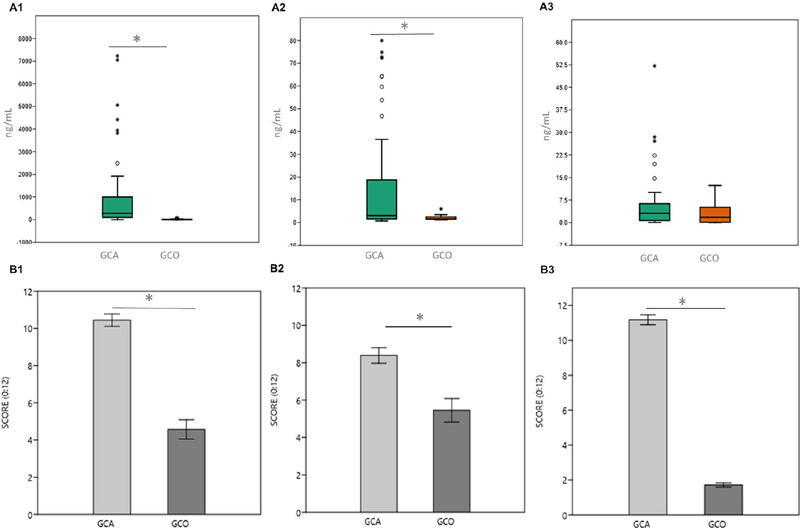
(A) Saliva concentration by ELISA cases vs. controls and (B) Tissue expression by immunohistochemistry cases vs. controls. Salivary concentrations were quantified using ELISA assays to detect A1) MMP-9 (Merck KGaA), A2) MMP-2 (Merck KGaA), and A3) TIMP-2 (Merck KGaA). Tissue expression levels were quantified by immunohistochemistry for B1) MMP-9 (Abcam), B2) MMP-2 (Abcam), and B3) TIMP-2 (Abcam). The levels and protein expressions in the control (GCO) and case groups (GCA) are shown. Results are expressed as means ± SD for each group. The salivary protein levels are shown in ng/mL (A, B, C), whereas tissue expression levels were calculated using SCORE (D, E, F). Statistically significant differences between groups are indicated by asterisks (p < 0.05).

A total of 60 saliva samples from OSCC cases and 30 from healthy controls were analyzed. MMP-9 concentration was significantly higher in the case group (936.47 ng/mL±207.17) than in controls (13.16 ng/mL±3.98) (p<0.05). Similarly, MMP-2 levels were markedly elevated in cases (79.93 ng/mL±14.98) in relation to controls (2.06 ng/mL±0.18) (p<0.05). In contrast, TIMP-2 concentrations failed to differ significantly between the two groups (cases: 5.60 ng/mL±1.11; controls: 2.99 ng/mL±0.63) (Figure 3).

In the bivariate analysis, a statistically significant association was observed between salivary MMP-2 concentrations and alcohol consumption frequency. Patients reporting lower alcohol intake (less than two days per week and fewer than five drinks per day) showed higher mean MMP-2 levels (17.07±3.50 ng/mL) than those with higher alcohol consumption (7.45±5.41 ng/mL; p = 0.03). However, lower mean TIMP-2 concentrations were observed in patients with lymphovascular invasion (2.25±1.24 ng/mL) than in those without lymphovascular invasion (6.04±1.27 ng/mL), with a borderline statistical significance (p = 0.06). No statistically significant associations were found between salivary MMP-9, MMP-2, or TIMP-2 concentrations and sex, age, tumor size, histological grade, lymph node involvement, extranodal extension, clinical stage, recurrence, mortality, smoking exposure, or comorbidities (Supplementary material).

To further evaluate the association between alcohol consumption and MMP-2 expression, a multiple linear regression model was performed using the MMP-2 score as the dependent variable and adjusting it for sex, smoking status, and age at diagnosis. After adjustment, alcohol consumption frequency showed no independent association with the MMP-2 score (β=1.043, p=0.363), indicating that the significant association in the bivariate analysis was confounded by the adjustment variables.

### MMP activity in saliva

**Figure 4:**
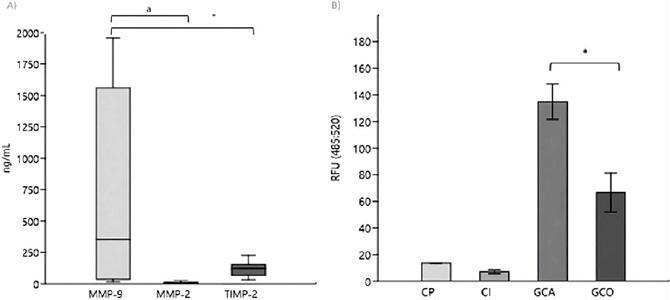
(A) Seric Elisa MMP in a subgroup of cases and (B) MMP activity in saliva cases vs. controls. The ELISA assay detected MMP-9 (Merck KGaA), MMP-2 (Merck KGaA), and TIMP-2 (Merck KGaA) to quantify serum concentrations. The fluorometric MMP activity assay (Abcam) quantified salivary MMP activity. The results show protein levels for the three biomarkers. They also describe activity levels in the positive (PC), inhibition control (IC), control (CGC), and case groups (CCG). The results of each group include means ± SD. Serum concentrations are shown in ng/mL, and activity results are expressed in relative fluorescence units (RFU). Asterisks (p < 0.05) and lowercase ‘a’ (p < 0.05) indicate statistically significant differences between groups.

MMP activity was assessed in saliva samples from 60 OSCC cases and 30 controls. Both groups showed higher MMP activity than the positive (13.61 RFU, ±0.07) and negative controls (7.02 RFU, ±1.52). The MMP activity in the case group (134.86 RFU, ±13.19) was significantly higher than in the control group (66.66 RFU, ±14.66) (p<0.05) (Figure 4). However, no significant correlation was observed between MMP activity and MMP-9 (p=0.244) or MMP-2 concentration (p=0.640).

### Expression of MMP-9, MMP-2, and TIMP-2 in tumor and normal tissue by immunohistochemistry

A total of 40 tissue samples were analyzed, including 20 tumor samples from OSCC cases and 20 samples of normal adjacent mucosa from the same patients. This normal adjacent tissue, located at least 1 cm from the tumor margins and histologically confirmed as free of neoplastic infiltration, was used as the control group for immunohistochemical comparisons.

Differences in the expression of MMP-2, MMP-9, and TIMP-2 were observed between OSCC tumor and control tissue (Figure 2). MMP-9 showed high-intensity expression (3+) in tumor cells, with a greater percentage of positive cells than in control mucosa, in which the expression showed lower intensity (1+ to 2+) and a smaller proportion of positive cells (Table 2). MMP-2 also showed strong intensity (3+) in tumor tissue, whereas control tissues showed minimal or no expression (0 to TIMP-2 expression differed between tumor and control tissue as well (Table 2).

**Table 2:** Expression of MMP-9, MMP-2, and TIMP-2 in tumor and healthy tissue.

**MMP-9 immunohistochemistry**									
**Normal tissue intensity staining**									
Nuclear epithelium		Cytoplasmic epithelium		Inflammatory cells		Stromal cells		Endothelial cells	
0+	3	0+	0	0+	0	0+	0	0+	0
1 +	15	1+	6	1+	0	1+	0	1+	0
2+	3	2+	15	2+	8	2+	12	2+	10
3+	0	3+	0	3+	14	3+	10	3+	12
**Proportion of staining normal tissue (%)**									
Nuclear Staining		Cytoplasmic Staining		Inflammatory cells		Stromal cells		Endothelial cells	
≤25%	4	≤25%	1	≤25%	0	≤25%	0	≤25%	0
26–50%	11	26–50%	13	26–50%	1	26–50%	0	26–50%	0
51–75%	0	51–75%	0	51–75%	0	51–75%	0	51–75%	0
>75%.	6	>75%.	7	>75%.	21	>75%.	22	>75%.	22
**Tumor tissue intensity staining**									
Nuclear		Cytoplasmic		Inflammatory cells		Stromal cell		Endothelial cells	
0+	4	0+	0	0+	0	0+	1	0+	0
1+	31	1+	0	1+	0	1+	0	1+	0
2+	4	2+	18	2+	15	2+	17	2+	18
3+	1	3+	22	3+	25	3+	22	3+	22
**Proportion of staining in tumor cells (%)**									
Nuclear		Cytoplasmic		Inflammatory cells		Stromal cell		Endothelial cells	
≤25%	4	≤25%	0	≤25%	0	≤25%	1	≤25%	0
26–50%	1	26–50%	0	26–50%	1	26–50%	0	26–50%	0
51–75%	0	51–75%	0	51–75%	0	51–75%	0	51–75%	0
>75%.	35	>75%.	40	>75%.	39	>75%.	39	>75%.	40
**MMP-2 immunohistochemistry**									
**Normal tissue intensity staining**									
Nuclear epithelium		Cytoplasmic Epithelium		Inflammatory Cells		Stromal Cells		Endothelial Cells	
0+	2	0+	0	0+	1	0+	0	0+	0
1+	6	1+	7	1+	1	1+	1	1+	2
2+	12	2+	15	2+	13	2+	13	2+	13
3+	3	3+	1	3+	6	3+	7	3+	6
**Proportion of staining normal tissue (%)**									
Nuclear Staining		Cytoplasmic Staining		Inflammatory Cells		Stromal Cells		Endothelial Cells	
≤25%	2	≤25%	2	≤25%	1	≤25%	0	≤25%	0
26–50%	7	26–50%	12	26–50%	0	26–50%	0	26–50%	0
51–75%	6	51–75%	1	51–75%	0	51–75%	0	51–75%	0
>75%.	8	>75%.	8	>75%.	20	>75%.	21	>75%.	21
**Tumor tissue intensity staining**									
Nuclear		Cytoplasmic		Inflammatory Cells		Stromal Cells		Endothelial Cells	
0+	0	0+	0	0+	0	0+	0	0+	1
1+	6	1+	0	1+	0	1+	0	1+	0
2+	29	2+	10	2+	23	2+	23	2+	20
3+	4	3+	29	3+	14	3+	15	3+	11
**Proportion of staining in tumor cells (%)**									
Nuclear		Cytoplasmic		Inflammatory Cells		Stromal Cells		Endothelial Cells	
≤25%	0	≤25%	0	≤25%	0	≤25%	0	≤25%	1
26–50%	0	26–50%	0	26–50%	1	26–50%	0	26–50%	0
51–75%	0	51–75%	0	51–75%	0	51–75%	0	51–75%	0
>75%.	39	>75%.	39	>75%.	36	>75%.	38	>75%.	31
**TIMP-2 immunohistochemistry**									
**Normal tissue intensity staining**									
Nuclear epithelium		Cytoplasmic Epithelium		Inflammatory Cells		Stromal Cells		Endothelial Cells	
0+	2	0+	0	0+	1	0+	0	0+	0
1 +	6	1+	7	1+	1	1+	1	1+	2
2+	12	2+	15	2+	13	2+	13	2+	13
3+	3	3+	1	3+	6	3+	7	3+	6
**Proportion of staining normal tissue (%)**									
Nuclear Staining		Cytoplasmic Staining		Inflammatory Cells		Stromal Cells		Endothelial Cells	
≤25%	0	≤25%	2	≤25%	1	≤25%	0	≤25%	0
26–50%	7	26–50%	12	26–50%	0	26–50%	0	26–50%	0
51–75%	6	51–75%	1	51–75%	0	51–75%	0	51–75%	0
>75%.	8	>75%.	8	>75%.	20	>75%.	21	>75%.	21
**Tumor tissue intensity staining**									
Nuclear		Cytoplasmic		Inflammatory Cells		Stromal Cells		Endothelial Cells	
0+	0	0+	0	0+	0	0+	0	0+	1
1+	6	1+	0	1+	0	1+	0	1+	0
2+	29	2+	10	2+	23	2+	23	2+	20
3+	4	3+	29	3+	14	3+	15	3+	11
**Proportion of staining in tumor cells (%)**									
Nuclear		Cytoplasmic		Inflammatory Cells		Stromal Cells		Endothelial Cells	
≤25%	0	≤25%	0	≤25%	0	≤25%	0	≤25%	1
26–50%	0	26–50%	0	26–50%	1	26–50%	0	26–50%	0
51–75%	0	51–75%	0	51–75%	0	51–75%	0	51–75%	0
>75%.	39	>75%.	39	>75%.	36	>75%.	23	>75%.	31

Expression of MMP-9, MMP-2, and TIMP-2 was also observed in inflammatory, stromal, and endothelial cells in tumor and control tissues (Table 2).

Given the ubiquitous labeling of these biomarkers, we used score values to compare mean cytoplasmic expression between tumor and control tissues, finding significant differences: MMP-9 (tumor: 10.44±0.32; control: 4.57±0.51) (p<0.05), MMP-2 (tumor: 8.38±0.42; control: 5.45±0.63) (p<0.05), and TIMP-2 (tumor: 11.17±0.28; control: 1.71±0.12) (p<0.05) (Figure 3).

The cytoplasmic expression of the biomarkers was evaluated regarding histopathological and sociodemographic variables using semi-quantitative scores. Higher TIMP-2 expression was observed in cases without lymphovascular invasion, with a median score of 12 (IQR: 12-12) when compared to cases with lymphovascular invasion, which showed a lower median score of 8 (IQR: 8–12), obtaining statistical significance (p<0.05). Moreover, MMP-9 expression was significantly higher in advanced clinical stages (III-IV), with a median score of 12 (IQR: 10–12) when compared to early stages (I–II), which showed a median score of 8 (IQR: 8–12) (p<0.05). A significant association was also observed between MMP-9 expression and alcohol consumption, with higher expression scores in patients reporting lower alcohol intake, showing a median score of 12 (IQR: 8-12) when compared to those with higher alcohol consumption, who showed a median score of 8 (IQR: 8–12) (p<0.05). No statistically significant differences were observed for MMP-2 expression across any evaluated variable and no significant associations were found between biomarker expression and smoking status (Supplementary material).

To further evaluate these findings and control for potential confounding, multiple linear regression models were performed adjusting for sex, smoking status, and age at diagnosis. In the model assessing TIMP-2 expression, lymphovascular invasion remained independently associated with the TIMP-2 score, explaining approximately 19.5% of its variability (adjusted R^2^=0.195). Patients with lymphovascular invasion showed, on average, lower TIMP-2 scores than those without invasion (B= −2.116, p=0.007), whereas sex, age, and smoking status showed no significant associations. Additionally, clinical stage remained independently associated with MMP-9 expression after adjustment, explaining approximately 21% of the variability in the MMP-9 score (adjusted R2=0.210), with patients in advanced stages (III–IV), showing higher scores than those in early stages (I–II) (B=2.092, p=0.003).

Based on the observed differences in immunohistochemical scores between tumor and control tissues, a post-hoc power analysis was performed for the main tissue comparisons. It showed high statistical power for detecting differences in MMP-9 (power=1.000), MMP-2 (power=0.975), and TIMP-2 (power=1.000) at a significance level of 0.05, supporting the adequacy of the sample size for the immunohistochemical analyses.

### Serum concentrations of MMP-9, MMP-2, and TIMP-2

Serum analysis was conducted in an exploratory manner in a subsample of 10 OSCC cases. The mean concentrations referred to MMP-9 (656.92±753.79 ng/mL), TIMP-2 (115.95±59.20 ng/mL), and MMP-2 (7.04±7.55 ng/mL) (Figure 4). As no serum sample from a control group was available, this analysis only descriptively characterized biomarker levels. Thus, no group comparisons or power calculations were performed, and these findings should be interpreted solely as exploratory observations without diagnostic or inferential implications.

### ROC curves, sensitivity, and specificity analysis of the techniques

ROC curves were used to assess the sensitivity, specificity, and optimal cut-off point for each protein in distinguishing between cases and controls.

**Figure 5:**
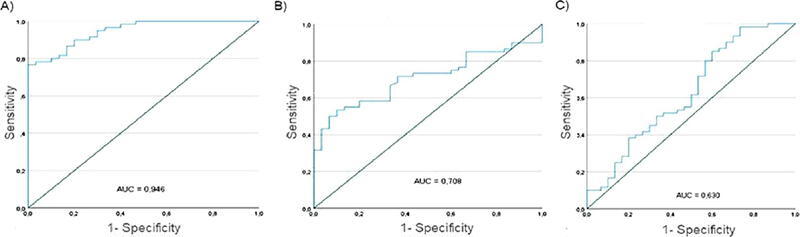
ROC curves for salivary MMP-9, MMP-2, and TIMP-2. Saliva concentrations measured by ELISA for A) MMP-9 (Merck KGaA), B) MMP-2 (Merck KGaA), and C) TIMP-2 (Merck KGaA). The ROC curves for the three proteins were calculated using saliva concentrations. The area under the curve (AUC) is provided for each protein.

Salivary MMP-9 showed excellent discriminatory capacity, with an optimal cut-off of 43.42 ng/ml (AUC=0.946, Sensitivity=0.817, 1-Specificity=0.133). MMP-2 also showed good discriminatory ability (AUC=0.708; cut-off=1.91 ng/ml, Sensitivity=0.717, 1-Specificity=0.367). TIMP-2 showed lower sensitivity (AUC=0.630; cut-off=1.62 ng/ml, Sensitivity=0.617, 1-Specificity=0.500) (Figure 5).

## Discussion

This study evaluated the potential of salivary concentrations of MMP-9, MMP-2, and TIMP-2 as biomarkers for liquid biopsy in OSCC. Its results showed significant differences in the salivary concentrations of MMP-2 and MMP-9 between the case and control groups, with a notable distinction in MMP activity as well.

Regarding serum biomarkers, analyses were performed in a small subsample of 10 OSCC cases without corresponding control samples. Therefore, these results cannot be used for direct case-control comparisons and should be considered strictly exploratory. The observed serological concentrations of MMP-9 in this limited subset support no diagnostic claims and lack sufficient statistical power for generalization. Future studies including matched serum samples from cases and controls with larger sample sizes are required to determine any potential clinical relevance.

This study used a 10 mL saline mouth rinse to collect salivary samples, which were then centrifuged and processed to obtain the supernatant for analysis. This procedure differs from standard stimulated or unstimulated saliva collection methods that are commonly described in the literature^15^, ^17^ and introduces an inherent dilution factor that may underestimate absolute protein concentrations and influence biomarker thresholds. However, comparison with previous studies indicates that the salivary concentration in this study lies within the reported ranges for oral rinse and unstimulated saliva. For example, salivary MMP-2 levels around 2.17±0.62 ng/mL in OSCC cases and salivary MMP-9 levels close to 49.27±44.50 ng/mL have been reported,^20^ values that are comparable to the cut-off points in this study (1.91 ng/mL for MMP-2 and 43.42 ng/mL for MMP-9). In recent years, oral rinse-based methodologies have been increasingly explored and validated in other oral and systemic inflammatory conditions, supporting their use as standardized and reproducible sampling approaches.^21^, ^22^ Nevertheless, the lack of direct validation against unstimulated saliva remains a methodological limitation that could have influenced the measured concentrations and downstream analyses.

ROC curve analysis was used to describe the discriminative performance of salivary MMP-2, MMP-9, and TIMP-2 in distinguishing OSCC cases from controls. The optimal cut-off values were 43.42 ng/mL for MMP-9 (AUC=0.946; sensitivity = 0.82; specificity = 0.87), 1.91 ng/mL for MMP-2 (AUC = 0.708; sensitivity = 0.72; specificity = 0.63), and 1.62 ng/mL for TIMP-2 (AUC = 0.630; sensitivity = 0.62; specificity = 0.50). While these values indicate a reasonable discriminatory capacity and are consistent with concentration ranges in previous salivary biomarker studies, the cut-off points are yet to be externally validated and should not be interpreted as definitive diagnostic thresholds. In clinical practice, ROC-derived cut-off values require validation in independent populations to establish their predictive value, including positive and negative predictive values. Accordingly, the ROC analysis in this study should be interpreted as exploratory and hypothesis-generating rather than confirmatory.

Various biomarkers have been proposed for OSCC diagnosis and prognosis, with particular sensitivity in distinguishing between healthy individuals and those with OSCC.^23^, ^24^ These biomarkers also hold promises for predicting the transformation of potentially malignant disorders.^25^ Among the key biomarkers (TNF-α, IL-1β, IL-6, IL-8, LDH), MMP-9 emerged as particularly valuable in differentiating between cases and controls, with an optimal cut-off point of 43.42 ng/ml by ELISA

The presence and activity of MMPs, such as MMP-2 and MMP-9, are crucial in tumor cell invasion and metastasis, processes that require the degradation of the ECM. This degradation is facilitated by protease enzymes, including serine proteases, cysteine proteases, and aspartic proteases.^26^ MMPs constitute a large family of zinc-dependent endopeptidases—classified into collagenases, gelatinases, stromelysins, and membrane-type MMPs, each with distinct substrate specificities and molecular weights (Figure 6). MMP-2 (gelatinase A) and MMP-9 (gelatinase B) were selected for this study because of their recognized role in degrading type IV collagen and gelatin, which are essential components of basement membrane remodeling and tumor invasion

**Figure 6:** Classification of matrix metalloproteinases (MMPs).

Subgroup	MMP	Nomenclature	Main Matrix Substrates
Interstitial collagenases	MMP-1	Fibroblast collagenase	Fibrillar collagen (III > I)
	MMP-8	Neutrophil collagenase	Fibrillar collagen (I > III)
	MMP-13	Collagenase-3	Fibrillar collagen
Gelatinases	MMP-2	Gelatinase A	Collagen I, IV, V; gelatin
	MMP-9	Gelatinase B	Collagen IV, V; gelatin
Stromelysins	MMP-3	Stromelysin-1	Laminin, fibronectin, non-helical collagen
	MMP-10	Stromelysin-2	Similar to MMP-3
	MMP-11	Stromelysin-3	Collagen IV, gelatin, laminin
	MMP-7	Matrilysin	Similar to MMP-3
Membrane-type MMPs (MT-MMPs)	MMP-14	MT1-MMP	Collagen I, II, III; gelatin; activates MMP-2
	MMP-15	MT2-MMP	
	MMP-16	MT3-MMP	Activates MMP-2
	MMP-17	MT4-MMP	
Elastase-type MMPs	MMP-12	Metalloelastase	Elastin, fibronectin

Furthermore, MMPs are involved in the degradation of the interstitial matrix of blood vessels and the generation of new blood vessels in the tumor microenvironment.^27^ This study found higher salivary concentrations of MMP-9 than of MMP-2, possibly due to MMP-2 constitutive expression in various tissues, whereas MMP-9 expression is more selectively induced by factors such as cytokines, growth factors, and cell-stroma interactions.^28^

Reports on the activation mechanisms of MMP-9 in tumor tissues indicate that the enzyme is typically found in its zymogen form.^29^ Proteases such as MMP-2, MMP-3, and MMP-13 have shown potential in vitro to activate MMP-9.^30^, ^31^ These findings from the literature suggest a possible link between MMP-2 concentration and MMP-9 activity in the ECM. MMP-2 and MMP-9 contribute to the degradation of the ECM and activate various signaling pathways that promote tumor progression.^32^ In the tumor microenvironment of OSCC, the overexpression of MMP-2 and MMP-9 is linked to signaling pathways such as mitogen-activated protein kinase and phosphoinositide 3-kinase/AKT.^33^ These pathways play crucial roles in metabolism, survival, and metastasis, which are key processes in cancer development.^34^ MMPs can also interact with cell surface receptors such as integrins and growth factor receptors, further enhancing signaling cascades that increase invasive potential and resistance to apoptosis in cancer cells.^35^, ^36^ The activation of these pathways by MMPs underscores their role only remodeling the tumor microenvironment and in directly influencing the behavior of tumor cells, making them more aggressive and capable of metastasis.^37^

The activation of MMP-2 involves complex mechanisms with MT-MMPs, such as MT1-MMP (MMP-14) playing a key role.^36^ MT1-MMP binds to the membrane-bound TIMP-2, which subsequently binds to the hemopexin domain of MMP-2, and another MMP-14 activates pro-MMP-2, which can remain bound or be released.^37^ Tissue expression of MT1-MMP might provide further insights into MMP-2 activity and the formation of MT1-MMP, TIMP-2, and MMP-2 complexes.

The regulation of MMP activity is intricately linked to TIMPs, particularly TIMP-2. The formation of MMP-TIMP complexes is essential for maintaining tissue homeostasis by controlling ECM degradation.^38^ Our study observed that, despite the elevated expression of MMPs in tumor tissues, TIMP-2 levels showed no significant differences between cases and controls in saliva. This could be attributed to the constitutive expression of TIMP-2 in tissues, which might enable cancer cells to bypass MMP regulation, leading to unchecked MMP activity that facilitates tumor progression. Moreover, the formation of these complexes may reduce the amount of active free MMPs, impacting the detectable levels of TIMP and MMPs in biological fluids such as saliva.

Our results suggest a potential relationship between MMP-9 concentrations in tissue and carcinoma stage, although this assessment is difficult to reproduce and thus might not be a recommended method. Immunohistochemistry analysis showed the overexpression of MMP-2 and MMP-9 in OSCC patients, with an association with advanced stage and histological grade.^12^, ^39^ However, the interpretation of immunohistochemistry results is complicated by the ubiquitous presence of these proteins in non-tumor and tumor tissue. The scoring system based on intensity and percentage better distinguished the groups, improving comparability across samples. Nonetheless, we recognize that this approach may still be affected by selection bias and potential overfitting. Therefore, the findings in this study should be interpreted with caution.

The use of normal adjacent mucosa as a control for immunohistochemistry represents an important limitation of this study. Although this approach is widely used due to tissue availability and patient-matched sampling, histologically normal tissue adjacent to carcinoma may harbor molecular alterations related to field cancerization, which can influence protein expression patterns and reduce the contrast between tumor and control tissue.^40^ However, from an ethical perspective, obtaining truly healthy oral tissue by biopsy is unfeasible as biopsies are performed exclusively for diagnostic or therapeutic purposes in the presence of disease. Consequently, the use of normal adjacent tissue has become an ethically accepted and pragmatic model in translational oral cancer research.

The immunohistochemistry analysis of MMP-2, MMP-9, and TIMP-2 showed differences between cases and controls when analyzed using a scoring system that grouped percentage with intensity.^19^ However, the widespread presence of these proteins in healthy and tumor tissue complicates interpretation. Despite these challenges, an association between MMP-9 expression and advanced carcinoma stage was observed that was consistent with previous studies.^12^ However, given the statistical limitations of this study and its absence of multivariable analysis to adjust for confounders, this result offers no evidence of prognostic value, only constituting a descriptive association.

All biomarkers were positively expressed in epithelial and tumor cells and in inflammatory, stromal, and endothelial cells. This is in line with previous reports that have described MMP-9 expression in stromal cells, such as fibroblasts, endothelial cells, and inflammatory cells.^41^, ^42^ This study also found that OSCC invasion and metastasis depend on tumor cell activity and on the interaction between neoplastic cells and the tumor microenvironment, which stimulates MMP expression in surrounding stromal cells.^41^, ^42^ These interactions stimulate MMP expression, promoting tumor cell invasion and the restructuring of the tumor microenvironment to support further tumor growth and spread.^43^ Stromal cells contribute equally to the degradation of the basement membrane just as tumor cells do. Moreover, they participate in other processes, such as promoting angiogenesis, inhibiting antitumor immune cells, and providing metabolites to tumor cells.^43^

Additionally, MMPs, particularly MMP-9, have pro-angiogenic properties, contributing to the formation of new blood vessels within the tumor microenvironment.^40^ This study observed high MMP-9 activity in tumor tissue and saliva, which is consistent with its role in promoting angiogenesis, a key process in tumor growth and metastasis. The ability of MMPs to modify the tumor vasculature highlights their dual role in supporting the physical spread of the tumor and in altering the microenvironment to favor tumor survival and expansion.^42^

Comparing immunohistochemistry and ELISA assays showed differences in TIMP-2 expression between the two techniques, which may be related to the formation of MMP-TIMP complexes that regulate MMP activity. The formation of activation complexes involving TIMP-2 could be associated with lower levels of free MMP in saliva.^44^

The inclusion and exclusion criteria in this study were defined by the HeadSpace project, which established a biobank of biological samples for research purposes (rather than specifically for this analysis). Accordingly, individuals with chronic systemic diseases associated with alcohol or tobacco consumption were excluded. However, the control group included individuals who reported alcohol or tobacco use, although without chronic comorbidities related to these habits. We acknowledge that this selection criterion may represent a limitation as it could partially affect the comparability with the OSCC group, in which smoking and alcohol use were frequent. These factors are well-known in OSCC pathogenesis and have been independently associated with elevated circulating levels of MMP-9, possibly by inflammatory and oxidative stress mechanisms.^45^ Therefore, future studies should include control populations with similar exposure profiles to better determine the independent contribution of these biomarkers to oral carcinogenesis.

An additional limitation related to control selection should be acknowledged. Although individuals with chronic systemic diseases associated with alcohol or tobacco use were excluded, this criterion fails to imply abstinence from alcohol or tobacco consumption among controls. Nevertheless, the control group may represent a population with a lower burden of tobacco-and alcohol-related inflammatory conditions than OSCC cases. Given that smoking and alcohol consumption are independently associated with increased MMP-9 levels via inflammatory and oxidative stress pathways^46^, ^47^, this imbalance may have contributed to an overestimation of the differences in salivary MMP-9 concentrations between cases and controls. This selection bias, inherent to the use of a biobank designed for multiple research purposes, limits the representativeness of the control group relative to the population at risk for OSCC and should be considered when interpreting the magnitude of biomarker differences.

In this study, the biological samples used for salivary and serological analyses were obtained from a biobank and stored at −80 °C for an average of three years before processing. Although this storage condition follows standardized biobanking protocols, prolonged freezing may influence the structural integrity and stability of certain proteins, particularly enzymatic molecules such as MMPs and TIMP-2. Previous studies have shown that salivary proteins and peptides may undergo partial degradation during long-term storage, with greater stability observed at −80 °C than at −20 °C, but with detectable alterations after one to two years of preservation.^48^, ^49^ Reports on salivary MMPs have shown relative stability under deep-freezing conditions but emphasize that storage duration may contribute to variability in absolute concentrations.^45^ Therefore, while the storage conditions in this study are supported by the literature, such prolonged storage should be considered a potential limitation that may have influenced the measured levels of MMP-2 and MMP-9. Future investigations should include dedicated stability assessments or comparisons with freshly collected samples to further validate biomarker robustness over time.

Therefore, storage duration represents a potential limitation of this study as it could have affected the absolute concentrations of MMP-2, MMP-9, and TIMP-2 that were detected in saliva. Future studies should include stability validation assays or analyses of freshly collected samples to confirm biomarker preservation over time.

This study highlights the potential relevance of salivary MMP-2 and MMP-9 concentrations to investigate OSCC-associated alterations. Its findings suggest that salivary MMP activity could be useful in distinguishing between cases and controls. In contrast, the interpretation of serum MMP-9 concentrations is limited by the small sample size and the absence of control samples in this research, which precludes definitive conclusions about its diagnostic value.

Although the ubiquitous presence of MMP-2, MMP-9, and TIMP-2 in healthy and tumor tissue complicates interpretation, analyzing their expression in tumor tissue and the surrounding microenvironment may provide valuable insights. However, the limited statistical power and sample size in this study restricts the generalizability of its findings and its ability to confirm non-significant results. These factors should be considered as important limitations this research. This study offers novel data on the concentrations and expressions of these biomarkers in OSCC for a Colombian cohort, emphasizing the need for future studies with larger sample sizes and multicenter participation.

## Conclusions

This study highlights the potential involvement of MMP-2 and MMP-9 in the pathogenesis and progression of OSCC. The higher salivary concentrations in OSCC patients when compared with controls suggest their possible relevance as exploratory biomarkers detectable by liquid biopsy. However, these findings should be interpreted with caution as they are based on an exploratory design, and prospective validation and comparative studies are still required.

The inclusion of a Colombian cohort provides valuable regional data, but the limited sample size and exploratory nature of the analyses in this study indicate that larger multicenter studies are needed to confirm these preliminary results and address the current limitations. This study also showed distinct expression patterns of MMP-2, MMP-9, and TIMP-2 in tumor tissues when compared to healthy tissues, with greater expression in OSCC cases. However, the ubiquitous expression of these proteins in tumor and healthy tissues necessitates the cautious interpretation of the results as it complicates the differentiation between pathological and normal states.

Despite these challenges, immunohistochemistry and ELISA provided descriptive evidence of differential expression, despite notable limitations. Additionally, the differences in MMP activity between the case and control groups suggest that MMPs play a role in ECM degradation without implying direct diagnostic applicability. The role of MMP-TIMP complexes in tissue homeostasis further underscores the complex regulatory mechanisms involved in OSCC progression.

Despite the promising findings, this study acknowledges the need for larger, multicenter studies to validate these observations before any clinical application can be considered. Future research should also explore the biological mechanisms underlying MMP activation and regulation in OSCC. Overall, this study provides valuable descriptive data on OSCC-associated MMP alterations and supports further investigation in well-powered validation studies.

## Data Availability

The datasets generated and analyzed during the current study are available in the SciELO Data repository - doi: 10.48331/SCIELODATA.CPZXC7.
